# Heterogeneity in risk and potential pathogenic associations of NAFLD among distinct prediabetic phenotypes in young and middle-aged adults

**DOI:** 10.3389/fendo.2026.1819674

**Published:** 2026-06-03

**Authors:** Baojia Zheng, Chan Li, Zhaoxiong Fang, Wenning Xu, Feina Xiao, Lili Su, Xiaoling Chen, Yali Xiang

**Affiliations:** The Fifth Affiliated Hospital of Sun Yat-sen University, Zhuhai, China

**Keywords:** machine learning algorithms, mediation analysis, non-alcoholic fatty liver disease (NAFLD), potential pathogenic associations, prediabetes

## Abstract

**Objective:**

Prediabetes exhibits a high comorbidity rate with non-alcoholic fatty liver disease (NAFLD), significantly increasing the risks of diabetes progression and other complications. This study aims to clarify the differences in NAFLD risk among different prediabetes phenotypes and explore their underlying associations.

**Methods:**

A cohort of 2127 prediabetic individuals aged 18–59 years was enrolled. Latent Profile Analysis (LPA) was employed to classify phenotypes based on age, insulin resistance score, and body mass index. Machine learning algorithms and mediation analysis were used to investigate the risk features of NAFLD and the phenotype-specific pathogenic pathways.

**Results:**

Four distinct prediabetes phenotypes were identified, each demonstrating significant differences in the probability of NAFLD. The prevalence of NAFLD was highest (87.93%) in Class 1, characterized by young individuals with severe insulin resistance and obesity. Conversely, the lowest prevalence (18.39%) was observed in middle-aged individuals with mild insulin resistance and normal weight. In Class 1 individuals, hepatocellular damage showed the strongest association with NAFLD. Young individuals with moderate insulin resistance and overweight exhibited a higher NAFLD risk associated with the activation of the metabolism-immunity-inflammation axis. Among middle-aged individuals with moderate insulin resistance and overweight, neutrophilic inflammation was the most relevant factor. Additionally, some individuals displayed exacerbated metabolic abnormalities associated with elevated triglycerides, indirectly increasing the risk of NAFLD.

**Conclusions:**

The study characterized novel prediabetes phenotypes and their pathogenic associations with NAFLD, providing a new perspective for targeted prevention and management. This research approach addresses the limitations of the “one-size-fits-all” management model and contributes to the improvement of endocrine disease management.

## 1 Introduction

Prediabetes is defined as a concentration of blood glucose below the diabetes threshold but above the normal level, which is associated with high risk of developing diabetes and other complications (1). The prevalence of prediabetes ranges from 35.7% to 50.1%, representing a significant public health challenge in China (2). However, the prediabetic population is not a homogeneous group, and its intrinsic phenotypes can lead to different disease risk trajectories (3). Non-alcoholic fatty liver disease (NAFLD), a common coexisting condition, affects around 38.32% of individuals with prediabetes (4). The comorbidity of prediabetes and NAFLD not only accelerates the onset of diabetes but also greatly increases the risk of cardiovascular disease (CVD), being one of the more common and fatal outcomes (5). The American Diabetes Association (ADA) updated its screening guidelines on June 24th, 2023, recommending universal screening for NAFLD in individuals with prediabetes (6).

Due to its high metabolic variability, prediabetes exhibit notable differences in NAFLD risk among individuals (7), particularly in younger and middle-aged groups. The early stage of metabolic disorders in this population is associated with an increased likelihood of accelerating the deterioration of dysglycemia (8). The diversity of prediabetes phenotypes significantly influenced NAFLD risk. Currently, the existing studies on the prediabetic phenotype have primarily focus on the changes in blood glucose, but alterations in body metabolism and age were more critical. Furthermore, the potential mediating associations linking specific prediabetes phenotypes to NAFLD remain unclear.

The objective of this study was to elucidate the risk disparities of NAFLD among distinct phenotypes of middle-aged and young individuals with prediabetes, along with the underlying pathogenic pathways. This research aims to provide a scientific foundation for targeted prevention and stratified intervention strategies for NAFLD, ultimately delaying the progression of type 2 diabetes mellitus and its associated comorbidities.

## 2 Methods

### 2.1 Study design and participants

A retrospective, cross-sectional study was conducted on young and middle-aged adults (18–59 years old) diagnosed with prediabetes. This study collected 2127 individuals through electronic health records (EHRs) from January 1, 2022 to December 31, 2023 at the Fifth Affiliated Hospital of Sun Yat-sen University. Prediabetes criteria included impaired fasting glucose (IFG) (5.6-6.9 mmol/L), abnormal HbA1c (5.7-6.4%), or 2-hour oral glucose tolerance test (OGTT) levels of 7.8-11.0mmol/L (IGT) (9). Exclusion criteria comprised age outside 18–60 year range and absence of abdominal ultrasound. Our study received approved from the Research Ethics Committee of the Fifth Affiliated Hospital of Sun Yat-sen University, Zhuhai, China (No.2026-K188-1). Due to the retrospective nature of the study, informed consent was waived by the ethics committee.

### 2.2 Definition of outcome

All enrolled patients underwent abdominal ultrasonography performed by an experienced ultrasound specialist. NAFLD was diagnosed based on the presence of at least two abnormal findings on abdominal ultrasonography, in accordance with the criteria for NAFLD assessment and management in China (10): increased liver echogenicity with deep ultrasound signal attenuation in the near-field region, liver tissue hyperechogenicity compared to the kidney cortex, and vascular blurring. Diagnosis of NAFLD necessitates the presence of fatty liver without excessive alcohol intake or other causes of chronic liver disease (11).

### 2.3 Covariables

Demographic information were gathered. The height and weight of the patients were measured by the health technicians at physical examination center. Body mass index (BMI) was calculated as weight (kg) divided by the square of height (m^2^). Blood pressure was measured at rest using a standardized protocol, and the mean systolic blood pressure (SBP) and diastolic blood pressure (DBP) were subsequently calculated. Hypertension was defined as SBP ≥ 140 mmHg and/or DBP ≥ 90 mmHg. Other variables measured included fasting blood sugar (FBG), HbA1c, total cholesterol (TC), triglyceride (TG), low-density lipoprotein-cholesterol (LDL-C), high-density lipoprotein-cholesterol (HDL-C), r-Glutamyl Transpeptidase (r-GT), albumin (ALB), total bilirubin (TBIL), white blood cell (WBC), platelet count (PLT), globulin (GLB), direct bilirubin (DBIL), total protein (TP), serum uric acid (SUA), monocyte (MONO), alanine aminotransferase (ALT), red blood cell (RBC), creatinine (Cr), hemoglobin (HGB), estimated glomerular filtration rate (eGFR), lymphocyte (LY), glutamic oxalacetic transaminase (AST), red cell distribution width-coefficient of variation (RDW-CV), red cell distribution width-standard deviation (RDW-SD) and neutrophil (NEUT). The other variables calculated as follows: Metabolic score for insulin resistance (METS-IR) =Ln[(2×FBG(mg/dL)+TG (mg/dL))× BMI/(Ln(HDL-C(mg/dL))] (12); Systemic inflammatory response index (SIRI) = neutrophil count × monocyte count/lymphocyte count (13); Hepatic steatosis index (HSI)= 8 ×(ALT/AST ratio)+BMI (+2, if female; +2, if diabetes mellitus) (14).

### 2.4 Study design

**Figure 1:**
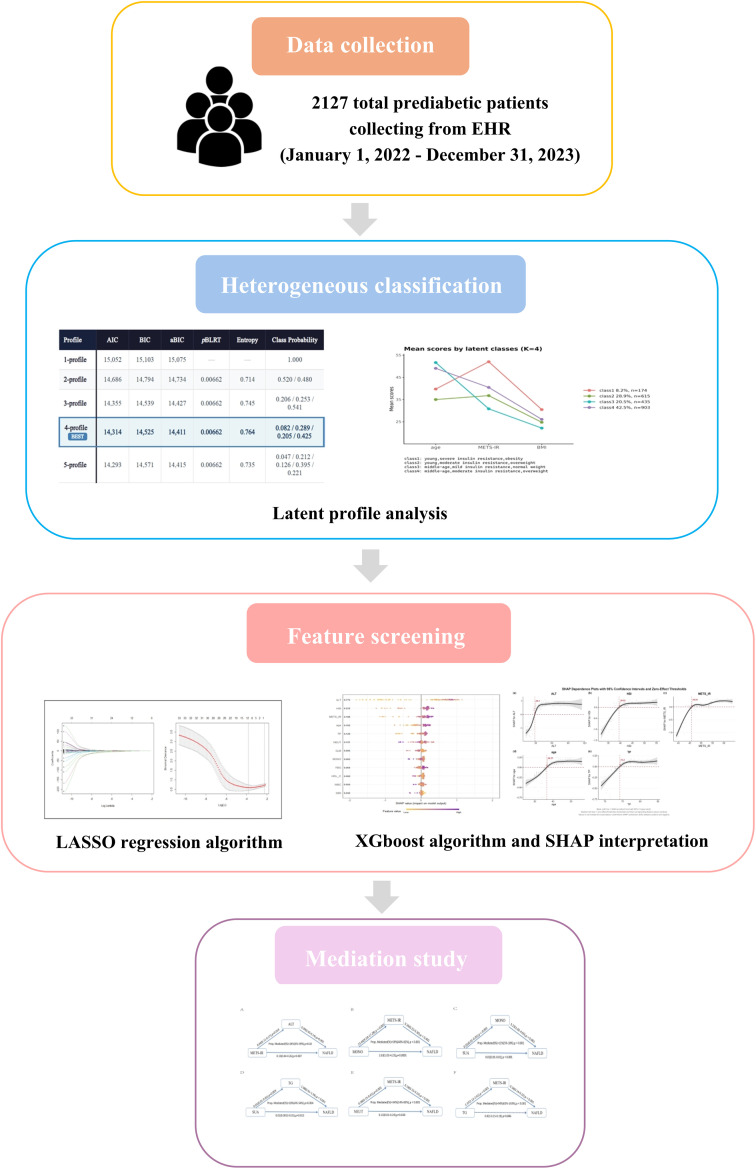
The flow of the study.

A total of 2127 prediabetic patients were enrolled from EHR between January 1, 2022 and December 31, 2023. Latent profile analysis (LPA) can detect hidden diversity in a population by categorizing individuals into different groups based on their responses to continuous variables (15). LPA was performed to classify prediabetes participants into subgroups based on three core indicators: age, BMI, and METS-IR. The optimal number of subgroups was determined by evaluating multiple fit indices, and the identified phenotypes were designated according to the distinct characteristics of age, BMI, and METS-IR. Subsequently, differences in the incidence of NAFLD were analyzed across the prediabetic phenotypes. Feature selection was implemented using the Least Absolute Shrinkage and Selection Operator (LASSO) regression algorithm. Extreme gradient boosting (XGBoost) models were constructed with LASSO-selected features, and Shapley Additive Explanation (SHAP) analysis was used for model interpretability. Finally, variables identified as the top five risk factors via SHAP analysis were considered candidate mediators, and mediation analysis was conducted to explore the potential association linking prediabetic phenotypes to NAFLD risk. More details were shown in **Figure 1**.

### 2.5 Statistical analysis

Statistical analysis was conducted using R software (version 4.4.3). All *p*-values were two-tailed, and statistical significance set at *p* < 0.05. The Chi-square test or Fisher exact probability method was employed to compare various grouping variables across distinct categories using the R*C table. The LAP analysis was conducted using the “mclust” package, fitting models with 1 to 5 latent profiles. Model fit was evaluated based on Bayesian Information Criterion (BIC), adjusted Bayesian (aBIC), Akaike Information Criterion (AIC), Entropy and Bootstrap Likelihood Ratio Test (BLRT). Lower AIC, BIC, and aBIC values indicate better model fit. Entropy, ranging from 0 to 1, reflects classification accuracy, with values closer to 1 suggesting higher accuracy. BLRT compared the fit of K-1 and K category models, with significant differences favoring the K-profile model when K represents the number of estimated parameters (16). LASSO regression was implemented using the “glmnet” and “foreign” packages. The optimal lambda (λ) value was determined via 10-fold cross-validation, selecting the value that minimized mean squared error. Variables with non-zero coefficients in the LASSO model were retained as candidate features for subsequent analysis. XGBoost models were constructed using the “xgboost” and “SHAPforxgboost” packages, with 5-fold cross-validation employed to evaluate model stability and avoid overfitting. SHAP analysis quantified the contribution of each feature to NAFLD risk and identified critical risk features for each prediabetic phenotype. Finally, mediation analysis was conducted using the R package “mediation” to assess mediation effects and potential mechanisms. Bootstrap resampling (1000 iterations) was incorporated to validate the robustness of the mediation effects.

## 3 Results

### 3.1 Study participants

A total of 2127 prediabetic patients were included in the study. The mean age of participants was 44 ± 8.34 years, with 1667 (78.4%) being males and 460(21.6%) female. The average body mass index (BMI) was 25.26 (standard deviation 3.32), while the mean of METS-IR was 38.39 (standard deviation 7.54). The average FBG level was 5.06 mmol/L (standard deviation 0.57), and the glycated hemoglobin level was 5.88% (standard deviation 0.22). 986 (46.4%) had comorbid NAFLD.

### 3.2 Latent profile analysis of prediabetes

**Figure 2:**
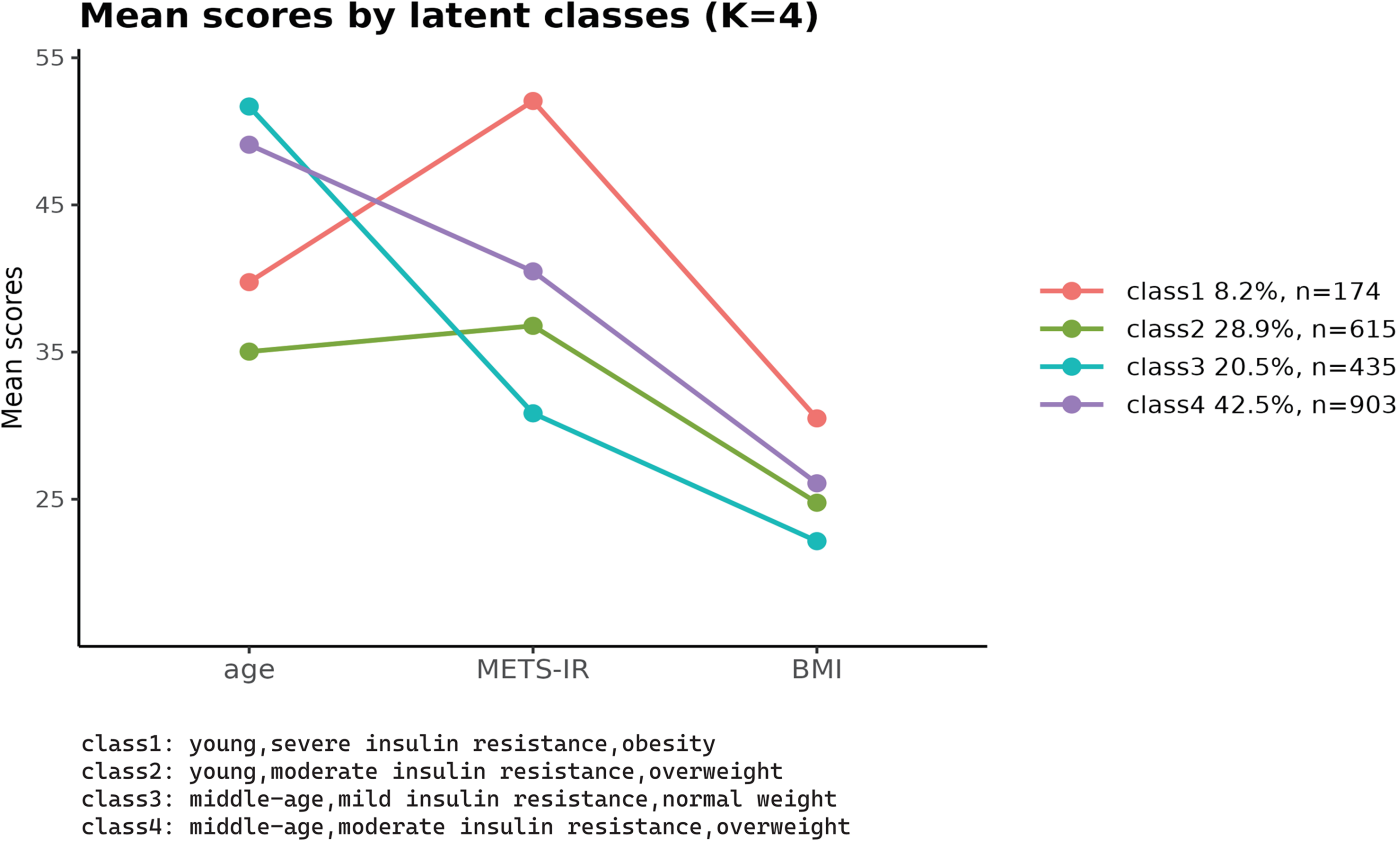
Latent profiles based on age, METS-IR and BMI in prediabetic individuals. METS-IR, metabolic score for insulin resistance. BMI, body mass index.

By incorporating age, METS-IR, and BMI as explicit variables, the estimation of latent profile numbers ranged from one to five. **Table 1** presented lower AIC, BIC, and aBIC values for the four-class pattern model compared to the three-class and five-class patterns, indicating a better model fit. The Entropy value for the four-class pattern model exceeded that of the three-class and five-class patterns, implying improved classification accuracy. Furthermore, BLRT demonstrated significant results (*p* < 0.001). Consequently, this study selected the four-class pattern model as the most suitable choice following a comprehensive assessment. 8.2% (174/2127) of patients were categorized as Class 1 (“young, severe insulin resistance obesity” phenotype), while 28.9% (615/2127) were classified as Class 2 (“young, moderate insulin resistance, overweight” phenotype). Additionally, 20.5% (435/2127) of patients were classified into Class 3 (“middle-age, mild insulin resistance, normal weight” phenotype). The remaining 42.5% (903/2127) of the patients were classified into Class 4 (“middle-age, moderate insulin resistance, overweight” phenotype). According to the average value of the explicit index, the latent profile was drawn, as shown in **Figure 2**.

**Table 1:** Fit indices for five models using latent profile analysis (N =2127).

Profile	AIC	BIC	aBIC	pBLRT	Entropy	Class Probability
1-profile	15052	15103	15075	–	–	1
2-profile	14686	14794	14734	0.00662	0.714	0.52/0.48
3-profile	14355	14539	14427	0.00662	0.745	0.206/0.253/0.541
**4-profile**	**14314**	**14525**	**14411**	**0.00662**	**0.764**	**0.082/0.289/0.205/0.425**
5-profile	14293	14571	14415	0.00662	0.735	0.047/0.212/0.126/0.395/0.221

AIC, Akaike information criterion; BIC, Bayesian information criterion; aBIC, sample-adjusted BIC; BLRT, bootstrap likelihood ratio test; Bold values indicated the best-fitting model based on model fit indices and theoretical interpretation.

### 3.3 LPA-based difference in potential variables and NAFLD

Statistically significant variations in baseline characteristics among the four classes were identified through ANOVA and Chi-square tests (all *p* < 0.001, **Table 2**), except for RDW-CV (*p* = 0.456). Further pairwise comparisons indicated that age was sequentially highest in Class 3, followed by Class 4, Class 1, and lowest in Class 2 (*p* < 0.001). METS-IR and BMI were sequentially highest in Class 1, followed by Class 4, Class 2, and lowest in Class 3 (*p* < 0.001). TG and SUA levels were sequentially highest in Class 1, followed by Class 4, Class 2, and lowest in Class 3 (*p* < 0.001), while HDL-C levels showed the opposite trend, being sequentially highest in Class 3, followed by Class 2, Class 4, and lowest in Class 1 (*p* < 0.001). More importantly, the prevalence of NAFLD was sequentially highest in Class 1, followed by Class 4, Class 2, and lowest in Class 3 (*p* < 0.001). Additional details were presented in **Table 2**.

**Table 2:** Baseline characteristic for four prediabetic classes.

Variables	Class 1 (N=174)	Class 2 (N=615)	Class 3 (N=435)	Class 4 (N=903)	X^2/^F	*p*
Age	39.75±0.57^a^	35.03±3.86^a^	51.68±4.13^a^	49.09±4.84^a^	1485.121	<0.001
METS-IR	52.06±6.62^a^	36.78±6.64^a^	30.84±3.35^a^	40.48±4.51^a^	767.946	<0.001
BMI(kg/m^2^)	30.49±3.91^a^	24.76±3.06^a^	22.15±1.72^a^	26.08±2.08^a^	505.015	<0.001
Gender(%)						
Male	160(91.95)^a^	479(77.88)^ab^	255(58.62)^ab^	773(85.60)^b^	147.004	<0.001
Female	14(8.05)^a^	136(22.12)^ab^	180(41.38)^ab^	130(14.4)^b^		
HbA1c(%)	5.89±0.22^a^	5.84±0.18^ab^	5.85±0.22^ab^	5.91±0.23^b^	2123	<0.001
FBG(mmol/L)	5.16±0.60^a^	4.95±0.49^ab^	5.01±0.58^ab^	5.14±0.59^b^	16.773	<0.001
TG(mmol/L)	3.82±3.54^a^	1.50±0.71^a^	1.23±0.49^a^	2.03±1.02^a^	189.328	<0.001
HDL-C(mmol/L)	0.98±0.27^a^	1.30±0.31^a^	1.57±0.34^a^	1.18±0.27^a^	218.08	<0.001
Hypertension(%)						
Yes	75(43.10)^a^	95(15.45)^ab^	91(20.92)^ab^	352(38.98)^b^	129.602	<0.001
No	99(56.9)^a^	520(84.55)^ab^	344(79.08)^ab^	551(61.02)^b^		
LDL-C(mmol/L)	2.87±0.89^a^	3.18±0.82^a^	3.36±0.94^a^	3.32±0.86^a^	16.40	<0.001
TC(mmol/L)	5.36±0.99^a^	5.23±0.88^bc^	5.61±1.03^abc^	5.46±0.96^c^	14.017	<0.001
SIRI	0.88±0.46^a^	0.74±0.64^a^	0.69±0.44^a^	0.78±0.46^a^	6.268	<0.001
r-GT(U/L)	60.77±44.81^a^	37.11±33.29^a^	29.83±29.68^a^	44.46±122.59^a^	6.723	<0.001
ALB(g/L)	47.82±2.91^a^	48.45±2.94^ab^	47.83±3.07^b^	47.61±2.93^b^	10.154	<0.001
TBIL(μmol/L)	10.31±4.77^a^	11.79±4.93^a^	11.82±4.91^a^	11.57±4.91^a^	4.661	0.003
WBC(*10^9^/L)	7.85±2.04^a^	6.78±1.75^a^	6.24±1.67^a^	6.95±1.68^a^	38.547	<0.001
PLT(*10^9^/L)	274.04±62.09^a^	265.68±61.13^b^	255.90±55.67^ab^	261.17±59.47^ab^	5.843	<0.001
GLB(g/L)	28.42±4.18^a^	27.64±4.09^a^	27.42±4.08^a^	27.11±4.13^a^	5.792	<0.001
DBIL(μmol/L)	3.75±1.47^a^	4.19±1.49^a^	4.20±1.50^a^	4.14±1.61^a^	4.107	0.006
TP(g/L)	76.25±3.93^a^	76.10±3.91^b^	75.25±4.04^ab^	74.71±4.21^ab^	17.189	<0.001
SUA(μmol/L)	451.13±111.42^a^	396.35±98.08^a^	359.71±91.18^a^	471.86±99.27^a^	49.743	<0.001
MONO(*10^9^/L)	0.48±0.13^a^	0.41±0.13^a^	0.39±0.14^a^	0.44±0.13^a^	27.544	<0.001
ALT(g/L)	40.91±24.65^a^	30.05±24.97^a^	20.86±16.13^a^	26.14±14.49^a^	50.225	<0.001
RBC(*10^12^/L)	5.32±0.44^a^	5.19±0.57^a^	4.93±0.53^a^	5.18±0.55^a^	30.719	<0.001
Cr(μmol/L)	86.39±13.81^a^	83.19±15.40^ab^	79.20±16.99^ab^	88.09±16.33^b^	33.216	<0.001
HGB(g/L)	155.91±10.86^a^	149.18±14.01^a^	143.79±13.90^a^	151.60±12.58^a^	48.705	<0.001
eGFR(ml/min)	95.23±14.62^a^	99.38±13.40^a^	89.17±13.17^a^	86.53±13.58^a^	118.798	<0.001
LY(*10^9^/L)	2.63±0.69^a^	2.32±0.61^a^	2.15±0.57^a^	2.38±0.64^a^	27.058	<0.001
AST(g/L)	25.05±10.64^a^	21.27±11.12^a^	20.73±13.36^a^	20.41±8.71^a^	9.453	<0.001
RDW-SD(fL)	42.52±2.82	42.13±2.76^a^	42.94±3.15^a^	42.93±3.30^a^	9.544	<0.001
RDW-CV(%)	13.13±0.94	13.19±1.16	13.24±1.15	13.26±1.22	0.869	0.456
NEUT(*10^9^/L)	4.45±1.54^a^	3.81±1.37^a^	3.49±1.24^a^	3.87±1.25^a^	23.088	<0.001
NAFLD(%)						
Yes	153(87.93)^a^	256(41.63)^a^	80(18.39)^a^	497(55.04)^a^	290.658	<0.001
No	21(12.07)^a^	359(58.37)^a^	355(81.61)^a^	406(44.96)^a^		

^a,b,c^The Least-Significant Difference showed statistical differences between two groups with the same letter.

FBG, fasting blood sugar; HbA1c, glycated hemoglobin; TC, total cholesterol; HDL-C, high-density lipoprotein-cholesterol; LDL-C, low-density lipoprotein-cholesterol; TG, triglyceride; r-GT, r-Glutamyl Transpeptidase; ALB, albumin; TBIL, total bilirubin; WBC, white blood cell; PLT, platelet count; GLB, globulin; DBIL, direct bilirubin; TP, total protein; SUA, serum uric acid; MONO, monocyte; ALT, alanine aminotransferase; RBC, red blood cell; Cr, creatinine; HGB, hemoglobin; eGFR, estimated glomerular filtration rate; LY, lymphocyte; AST, glutamic oxalacetic transaminase; RDW-SD, red cell distribution width-standard deviation; RDW-CV, red cell distribution width-coefficient of variation and NEUT, neutrophil. METS-IR, metabolic score for insulin resistance; SIRI, systemic inflammatory response index; NAFLD, non-alcoholic fatty liver disease.

### 3.4 Screening and importance ranking of NAFLD risk features

**Figure 3:**
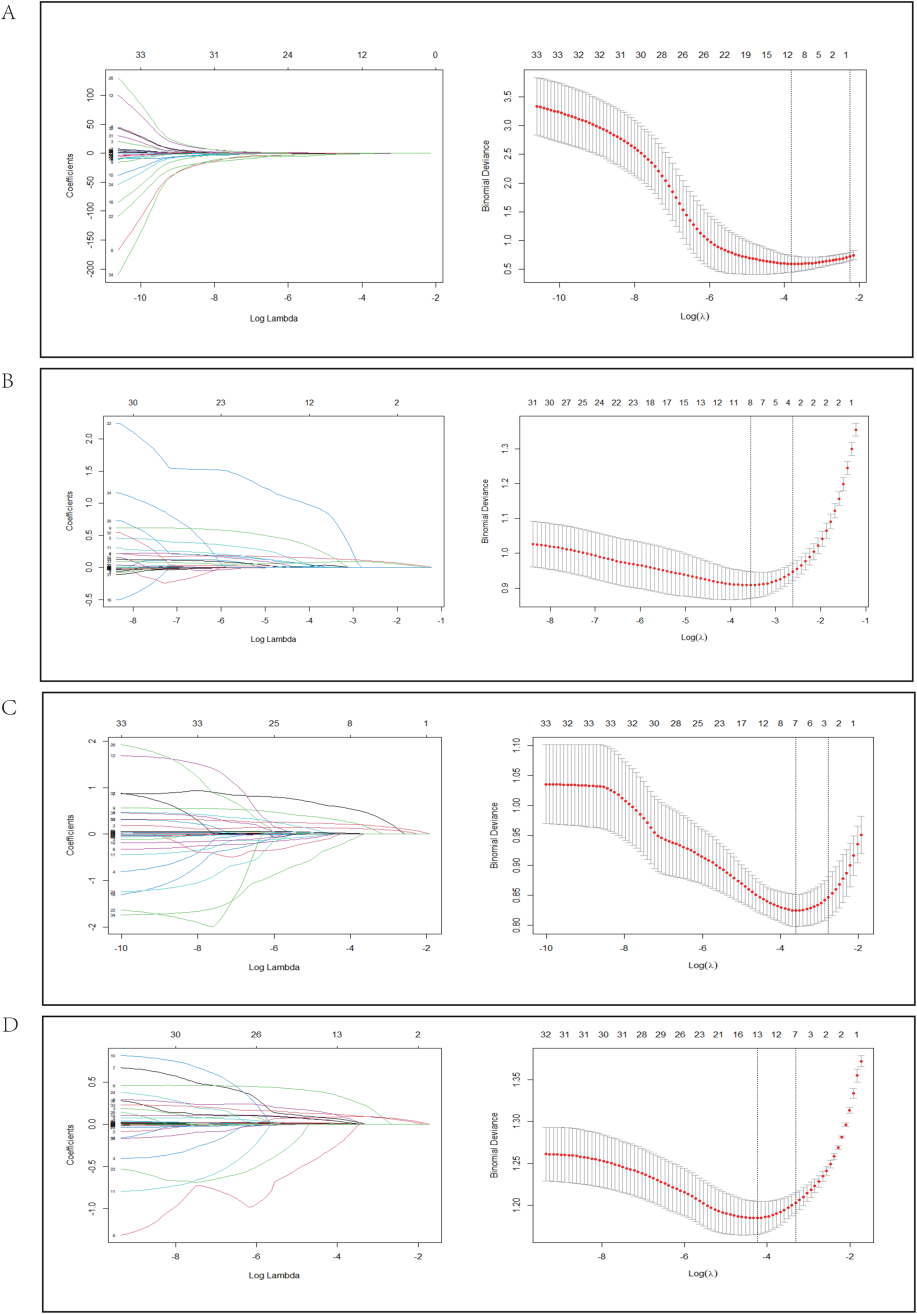
LASSO regression coefficient profiles and cross-validation curves for feature selection in each prediabetes phenotype class. For each class [**(A)** Class 1, **(B)** Class 2, **(C)** Class 3, **(D)** Class 4], the left panel showed the coefficient paths of variables as the Log(λ) penalty decreases, where each colored line represented a predictor variable, and the numbers at the top indicated the number of non-zero coefficients at differentλvalues. The right panel displayed the cross-validated binomial deviance curve, with the red dotted line representing the mean deviance and the gray error bars indicating standard error. The vertical dashed lines mark the optimalλvalues (lambda.min and lambda.1se) selected via 10-fold cross-validation, which balanced model fit and parsimony.

**Figure 4:**
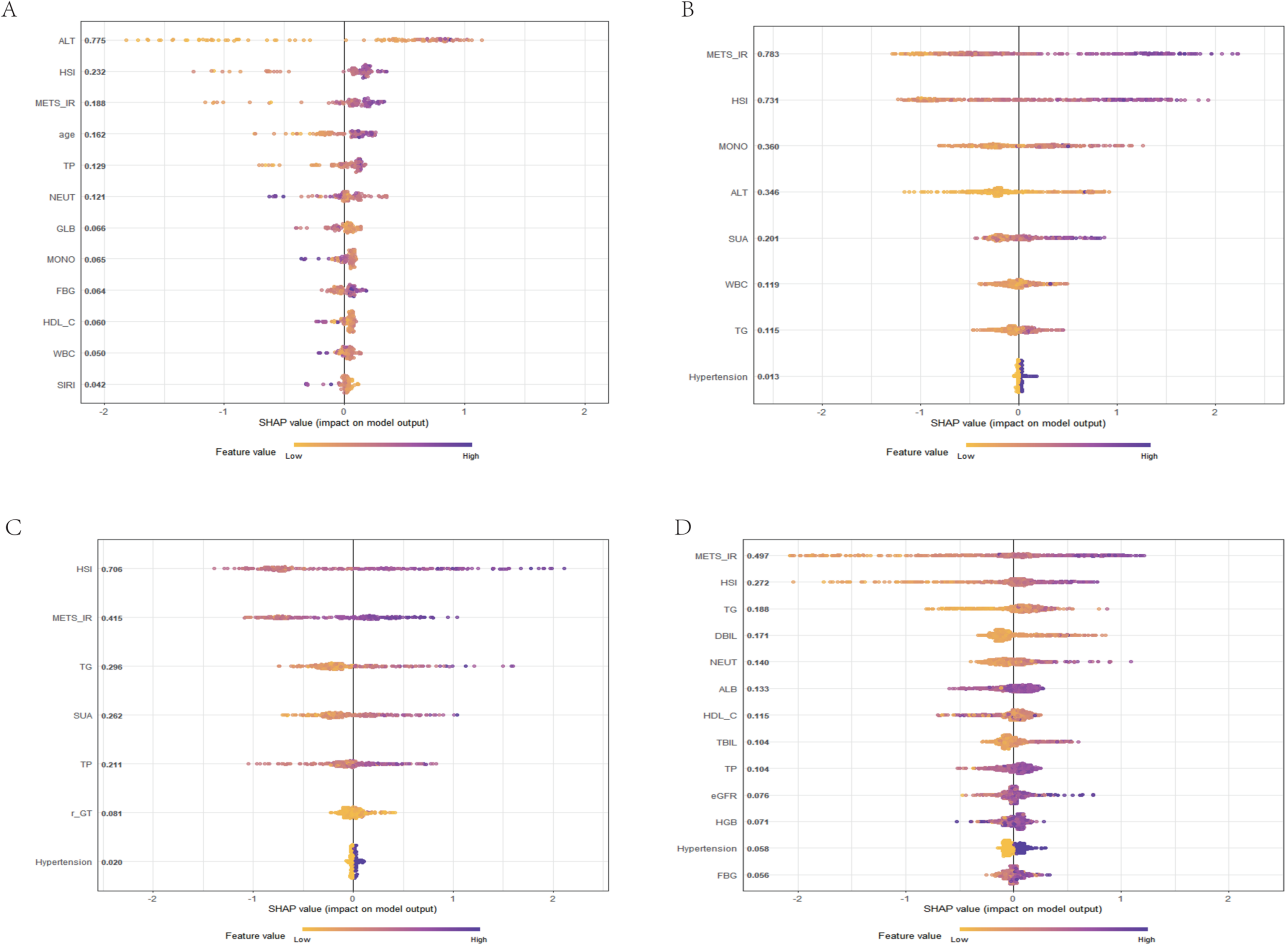
SHAP value models feature importance analysis. The impact of each feature was demonstrated on prediction results in the XGBoost model. The horizontal axis represented the SHAP values. The higher the SHAP value of a feature, the higher the probability of NAFLD. **(A)** Ranking of significant features linked to the presence of NAFLD in Class 1. **(B)** Ranking of significant features linked to the presence of NAFLD in Class 2. **(C)** Ranking of significant features linked to the presence of NAFLD in Class 3. **(D)** Ranking of significant features linked to the presence of NAFLD in Class 4. FBG, fasting blood sugar; HDL-C, high-density lipoprotein-cholesterol; TG, triglyceride; r-GT, r-Glutamyl Transpeptidase; WBC, white blood cell; ALB, albumin; TBIL, total bilirubin; GLB, globulin; DBIL, direct bilirubin; TP, total protein; SUA, serum uric acid; MONO, monocyte; ALT, alanine aminotransferase; HGB, hemoglobin; eGFR, estimated glomerular filtration rate; NEUT, neutrophil; METS-IR, metabolic score for insulin resistance; SIRI, systemic inflammatory response Index; HSI, hepatic steatosis index; non-alcoholic fatty liver disease, NAFLD.

To accurately identify the specific linking factors of NAFLD in each prediabetic phenotype, the LASSO regression algorithm was used for feature screening (**Figure 3**), effectively eliminating multicollinearity interference. Consequently, Class 1 yielded 12 characteristic variables, and Class 4 yielded 13, indicating that more complex pathogenic pathways in high-risk phenotypes. Class 2 yielded 8 features, while Class 3 yielded only 7 features, consistent with relatively singular associating factors in the low-risk population. Further, the contribution of each significant feature to the risk of NAFLD was quantified through XGBoost model construction combined with SHAP interpretation. And the result of SHAP analysis were as follow:

Class 1 (**Figure 4A**): The top 5 core characteristics were ALT (mean SHAP value of 0.775 ± 0.030, 95%CI: 0.714-0.838), HSI (mean SHAP value of 0.232 ± 0.021, 95%CI: 0.194-0.276), METS-IR (mean SHAP value of 0.188 ± 0.020, 95%CI: 0.153-0.234), age (mean SHAP value of 0.162 ± 0.011, 95%CI: 0.141-0.184), and TP (mean SHAP value of 0.129 ± 0.012, 95%CI: 0.107-0.154). It was worth noting that ALT surpassed HSI and METS-IR, suggesting hepatocyte injury as a core related factor in the development of NAFLD.

Class 2 (**Figure 4B**): The top 5 core features were METS-IR (mean SHAP value of 0.783 ± 0.023, 95%CI: 0.741-0.826), HSI (mean SHAP value of 0.731 ± 0.020, 95%CI: 0.693-0.773), MONO (mean SHAP value of 0.36 ± 0.010, 95%CI: 0.341-0.380), ALT (mean SHAP value of 0.346 ± 0.011, 95%CI:0.324-0.369), and SUA (mean SHAP value of 0.201 ± 0.008, 95%CI: 0.186-0.217). MONO as a biomarker of inflammatory cells, had a SHAP value second only to METS-IR and HSI, highlighting the critical role of immune inflammation in this phenotype.

Class 3 (**Figure 4C**): The top 5 core features were HSI (mean SHAP value of 0.706 ± 0.022, 95%CI: 0.663-0.749), METS-IR (mean SHAP value of 0.415 ± 0.016, 95%CI: 0.384-0.447), TG (mean SHAP value of 0.296 ± 0.013, 95%CI: 0.271-0.322), SUA (mean SHAP value of 0.262 ± 0.011, 95%CI: 0.241-0.284), and TP (mean SHAP value of 0.211 ± 0.012, 95%CI: 0.187-0.233). The SHAP values of each feature indicated that this phenotype of NAFLD result from the minor contributions of multiple factors.

Class 4 (**Figure 4D**): The top 5 core features were METS-IR (mean SHAP value of 0.497 ± 0.020, 95%CI: 0.456-0.536), HSI (mean SHAP value of 0.272 ± 0.013, 95%CI: 0.246-0.297), TG (mean SHAP value of 0.188 ± 0.007, 95%CI: 0.176-0.201), DBIL (mean SHAP value of 0.171 ± 0.006, 95%CI: 0.159-0.182), and NEUT (mean SHAP value of 0.140 ± 0.005, 95%CI: 0.130-0.150). The result revealed that lower DBIL and higher NEUT levels predicted NAFLD. Further details were provided in **Supplementary Table 1**.

### 3.5 The dose-response relationship between the key features of each phenotype and the risk of NAFLD

**Figure 5:**
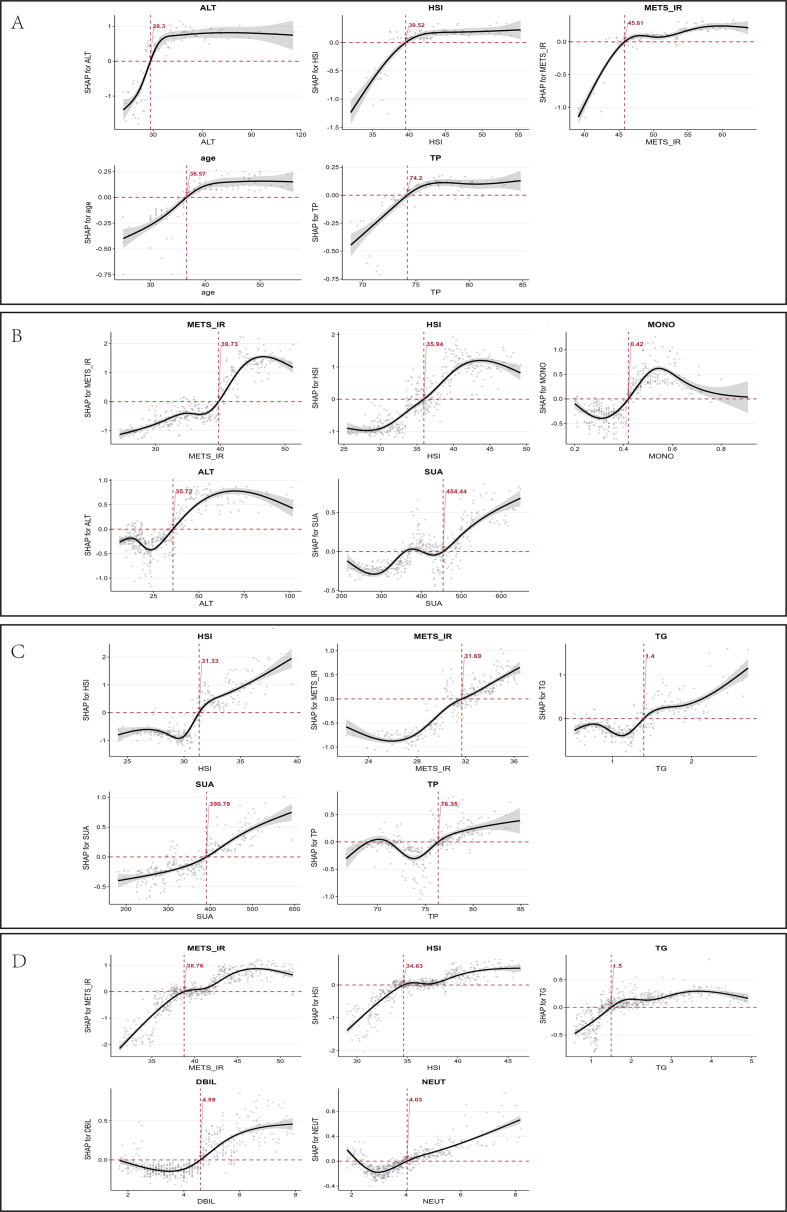
SHAP dependence plots with 95% confidence Intervals and thresholds in four classes. **(A)** The plot demonstrated the contribution of five top features to NAFLD predictions in Class 1. **(B)** The plot demonstrated the contribution of five top features to NAFLD predictions in Class 2. **(C)** The plot demonstrated the contribution of five top features to NAFLD predictions in Class 3. **(D)** The plot demonstrated the contribution of five top features to NAFLD predictions in Class 4. SHAP, SHapley Additive explanations. The grey area indicated the 95% confidence interval. TG, triglyceride; ALB, albumin; DBIL, direct bilirubin; TP, total protein; SUA, serum uric acid; MONO, monocyte; ALT, alanine aminotransferase; NEUT, neutrophil; METS-IR, metabolic score for insulin resistance; HSI, hepatic steatosis index; non-alcoholic fatty liver disease, NAFLD.

This study utilized the SHAP feature dependency plot to illustrated the dose-dependent association between core features of four prediabetic phenotypes and the risk of NAFLD. For ALT, a critical predictor in Class 1, the SHAP value demonstrated a clear positive association with ALT levels. As ALT increased beyond 28.3 U/L, the SHAP value transitioned from negative to positive, highlighting a significant non-linear dose-dependent relationship where elevated ALT levels markedly increased NAFLD risk in this phenotype (**Figure 5A**). Within Class 2, the relationship between MONO, SUA and NAFLD risk exhibited more pronounced dose-response characteristics. Elevated MONO levels exceeding 0.42×10^9^/L and SUA levels surpassing 454 umol/L correspond to increased SHAP values and heightened NAFLD risk (**Figure 5B**). Class 3 revealed a notable positive dose-dependent relationship among HSI, METS-IR, SUA, and TG, underscoring the phenotype’s complexity stemming from the cumulative interplay of multiple factors (**Figure 5C**). In Class 4, METS-IR and NEUT levels exhibited a significant positive dose-effect correlation with SHAP values. Notably, SHAP values gradually increased as NEUT counts exceed 4.03×10^9^/L, indicating a dose-dependent pathogenic impact of neutrophil inflammation. Moreover, a slight positive dose-dependent relationship with SHAP values was observed when TG levels surpassed 1.5 mmol/L (**Figure 5D**).

### 3.6 Exploratory mediating associations for NAFLD across the four prediabetic phenotypes

**Figure 6:**
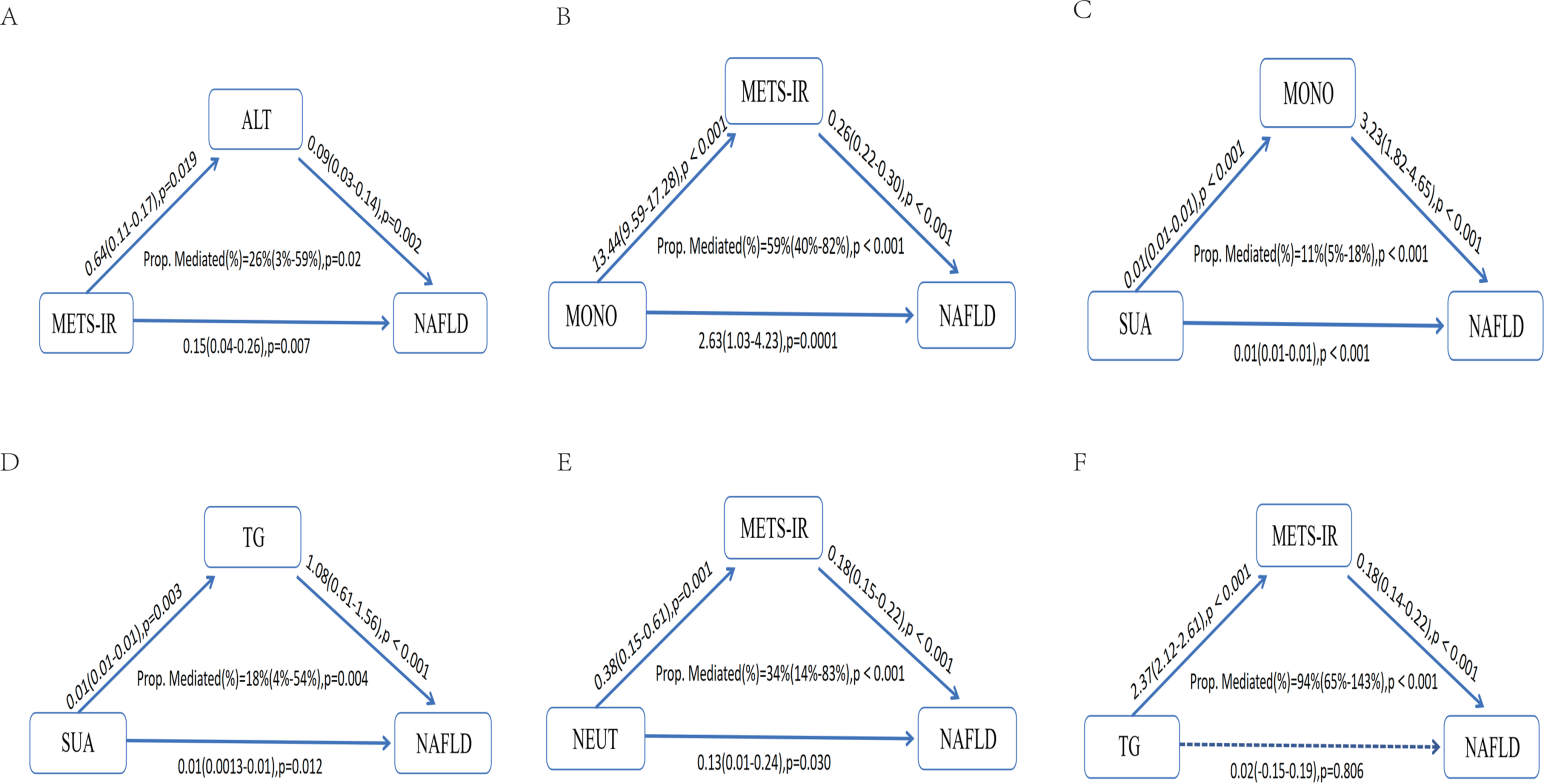
The mediating mechanism of NAFLD pathogenesis in four prediabetic classes. **(A)** The mediating analysis of NAFLD pathogenesis in Class 1. **(B, C)** The mediating analysis of NAFLD pathogenesis in Class 2. **(D)** The mediating analysis of NAFLD pathogenesis in Class 3. **(E, F)** The mediating analysis of NAFLD pathogenesis in Class 4. METS-IR, metabolic score for insulin resistance; ALT, alanine aminotransferase; MONO, monocyte; SUA, serum uric acid; TG, triglyceride; NEUT, neutrophil; NAFLD, non-alcoholic fatty liver disease.

Age, gender, and BMI were included as covariates in the mediation analyzes for the four prediabetic classes. **Figure 6A** showed the mediation analysis of Class 1. When METS-IR and ALT were simultaneously entered into the regression model to predict NAFLD, both METS-IR and ALT significantly and positively predicted NAFLD (β=0.64, *p* = 0.019; β=0.09, *p* = 0.002). ALT partially mediated the effect of METS-IR on NAFLD, with a mediated proportion of 26% (95%CI: 3%-59%, *p* = 0.02), confirming the completeness of the mediation pathway (METS-IR→ALT→NAFLD).

MONO-SUA-characterized activation of “metabolism-immunity-inflammation” axis showed strong associations with NAFLD in Class 2, as depicted in **Figures 6B, C**. MONO was associated with NAFLD via METS-IR with a mediated proportion of 59% (95%CI: 40%-82%, *p* < 0.001), while SUA was associated with NAFLD via MONO with a mediated proportion of 11% (95%CI: 5%-18%, *p* < 0.001).

The mediation analysis of Class 3 was presented in **Figure 6D**. TG significantly and positively predicted NAFLD (β= 1.08, 95%CI: 0.61-1.56, *p* < 0.001); Moreover, SUA predicted NAFLD through TG, with a mediated proportion of 18% (95%CI: 4%-54%, *p* = 0.004).

The mediation analysis of Class 4 was depicted in **Figures 6E, F**. Notably, NEUT exhibited both the direct and indirect effects on the presence of NAFLD in patients with Class 4. In contrast to Class 3, TG showed no direct association with NAFLD but was linked to NAFLD via METS−IR mediation, with a mediated proportion of 94% (95%CI: 65%-143%, *p* < 0.001).

## 4 Discussion

This study identified four novel metabolic phenotypes with significant heterogeneity in young and middle-aged prediabetic populations. Furthermore, each phenotype exhibited distinct specificity regarding the risk of NAFLD and its core associated factors, offering a novel perspective for the precise prevention and control of NAFLD.

The heterogeneity of prediabetes phenotypes is of critically significance for clinical stratified management. A prior study examining phenotypic heterogeneity identified four distinct phenotypes based on 12 clinical variables, revealing variations in the risks of type 2 diabetes (T2DM), chronic kidney disease (CKD), and cardiovascular disease (CVD) (17). Consistently, our study demonstrated differential NAFLD risk across these four phenotypes. It was found that the prevalence of NAFLD in young individuals with severe insulin resistance and obesity was as high as 87.93%, identifying them as an extremely high-risk group, compared to 18.39% in middle-aged individuals with mild insulin resistance and normal weight. This risk gradient strongly correlated with the severity of the metabolic phenotype. Recent research indicates that positive lifestyle modifications and weight loss are most effective for glycemic control in patients with prediabetes and fatty liver (3). Integrating our research findings, it is clear that being overweight or obese significantly accelerates the progression of diabetes in prediabetic patients with fatty liver. Given that insulin resistance is the central pathological mechanism of NAFLD (18), the combination of severe insulin resistance and obesity exacerbate metabolic disorders and accelerated the development of NAFLD (19). Notably, under comparable metabolic conditions of moderate insulin resistance and overweight, middle-aged individuals exhibited a significantly higher NAFLD risk than younger individuals. This suggests that the age-related decline in hepatic metabolic function, compounded by the accumulation of chronic inflammation, may potentiate the pathogenic effects of metabolic disorders (20). This observation enhanced our understanding of the interplay between age and metabolic factors in relation to NAFLD and addressed the limitations of prior research.

This study explored the phenotype-related pathogenic patterns of NAFLD in individuals with prediabetes. In young individuals with severe metabolic abnormalities, NAFLD was primarily associated with insulin resistance and hepatocyte damage (ALT-mediated). ALT, a specific functional enzyme locates in the cytoplasm of liver cells, serves as a direct indicator of liver cell damage when its serum levels are elevated. This enzyme is released in significant quantities into the bloodstream only when the integrity of the liver cell membrane is compromised (21). Severe insulin resistance induces the liberation of free fatty acids from adipose tissue, leading to heightened lipid synthesis and deposition in the liver. However, excessive fat accumulation within hepatic cells causes direct damage to cell membrane integrity, elevating the secretion of ALT. ALT release can exacerbate liver cell injury by triggering inflammatory signaling pathways, establishing a detrimental cycle (22). These findings underscored the importance of implementing advanced hepatoprotective strategies and insulin resistance amelioration for individuals with higher ALT in this cohort.

For young individuals with moderate metabolic abnormalities, MONO could directly and indirectly promote NAFLD. The mediating effect within the MONO→METS-IR→NAFLD pathway accounts for 59%, underscoring the distinct role of immune-inflammation in the young population. MONO serve as primary precursors of liver macrophages, migrating to the liver and differentiating into inflammatory macrophages in response to signals associated with metabolic disorders. These inflammatory macrophages activate hepatic stellate cells (HSCs) by releasing pro-inflammatory factors and signaling molecules (23). Prior research suggested that HSCs play a key role in inducing insulin resistance via their inflammatory characteristics and are strongly associated with the advancement of diabetes and NAFLD (24). Additionally, elevated levels of uric acid can trigger monocytes, resulting in inflammation and exacerbating insulin resistance (25). This study demonstrated that MONO and SUA collaboratively established the “metabolism-immune-inflammation” axis in Class 2 phenotype, playing a significant role in the comorbidity of NAFLD. This finding provides the epidemiological evidence for the monitoring of SUA and exploring anti-inflammatory strategies in these patients.

Among middle-aged overweight prediabetic individuals, neutrophils comprised 34% of the risk associated with insulin resistance-related NAFLD, underscoring neutrophilic inflammation as a key contributing factor in this group. In states of aberrant weight and metabolism, neutrophils may aggregate in insulin-sensitive tissues like the liver, inducing hepatic insulin resistance and subsequently initiating NAFLD (26). Neutrophil count can serve as a novel inflammatory biomarker for predicting fatty liver risk in individuals with Class 4 phenotype, aiding in the differentiated evaluation of conventional metabolic parameters. Consistent with recent mechanistic findings in type 2 diabetes mellitus (T2DM), elevated inflammatory markers exacerbate insulin resistance, promoting a vicious cycle of metabolic and immune dysregulation (27). Notably, TG serve as a crucial detection parameter for these prediabetic patients, with TG affecting NAFLD through insulin resistance. The excessive accumulation of triglycerides in the liver is a fundamental event in the onset and advancement of NAFLD, disrupting the liver’s lipid equilibrium and exacerbating insulin resistance (28). Besides, metabolic irregularities exhibit a stronger association with age in overweight and obese individuals (29). Therefore, concurrent targeted inflammatory intervention and lipid management should be implemented in this prediabetic cohort to improve glycemic control and delay the onset and advancement of NAFLD.

This study has several limitations that should be acknowledged. First, the cross-sectional design precluded determination of temporal order and causal inference. All mediation findings were exploratory. Second, NAFLD was diagnosed solely by abdominal ultrasound, which has reduced sensitivity for mild hepatic steatosis and may lead to slight underestimation of its true prevalence. Third, the study population was derived from EHR data of a single regional setting. External validation in multi-center was necessary to confirm the stability and applicability of the identified phenotypes. Furthermore, the important potential confounders, including dietary patterns, physical activity, alcohol consumption, genetic polymorphisms such as PNPLA3, and gut microbiota were not available in the EHR dataset. The lack of these variables could introduce residual confounding and influence the stability and interpretability of feature importance derived from SHAP analysis. Last but not the least, the applicability of four phenotypes in different ethnic groups, rural populations, or elderly populations remains unclear. Histological confirmation of NAFLD and liver fibrosis staging were not available, limiting the clinical interpretability of disease severity. Additionally, the study cohort exhibited marked male predominance, limiting the generalizability of the findings to female populations and introducing potential estimation bias. However, a rigorous methodological method was adopted for phenotype identification and mechanism exploration. The four-classification model employed demonstrated strong goodness of fit, with a balanced proportion across each category, thereby satisfying the criteria for clinical stratification. Additionally, the core associated factors for each phenotype were elucidated through a combined model of multiple machine learning algorithm, supporting the robustness of the exploratory pathway analysis. This study provides novel, clinically relevant insights into phenotype−specific associated pathways underlying NAFLD. Furthermore, the phenotype-oriented precise strategy effectively tackled the inefficiencies associated with one-size-fits-all intervention measures, presenting a viable solution for enhancing the prevention and treatment of NAFLD in prediabetic individuals.

## 5 Conclusion

This study clarified the heterogeneity and pathogenic associations of NAFLD across the prediabetic phenotypes, establishing a scientific foundation for the precise prevention and stratified intervention of NAFLD in prediabetic individuals. This approach addressed the limitations of a “one-size-fits-all” management, thereby improving the clinical outcomes of metabolic diseases.

## Data Availability

The raw data supporting the conclusions of this article will be made available by the authors, without undue reservation.
