# Supplementary material for: Heterogeneity in risk and potential pathogenic associations of NAFLD among distinct prediabetic phenotypes in young and middle-aged adults
Source: Front Endocrinol (Lausanne). 2026 Jun 3;17:1819674. doi: 10.3389/fendo.2026.1819674 (PMC13271963; doi:10.3389/fendo.2026.1819674)
Supplement: Supplementary file 1 [file Table1.docx]

**Supplementary Table 1** SHAP values of the specific features in prediabetic classes.

| Categories | Feature | Mean of SHAP | SD | CI_lower | CI_upper |
| --- | --- | --- | --- | --- | --- |
| Class 1 | ALT | 0.775 | 0.030 | 0.714 | 0.838 |
|  | HSI | 0.232 | 0.021 | 0.194 | 0.276 |
|  | METS_IR | 0.188 | 0.020 | 0.153 | 0.234 |
|  | age | 0.162 | 0.011 | 0.141 | 0.184 |
|  | TP | 0.129 | 0.012 | 0.107 | 0.154 |
|  | NEUT | 0.121 | 0.013 | 0.097 | 0.146 |
|  | GLB | 0.066 | 0.006 | 0.055 | 0.079 |
|  | MONO | 0.065 | 0.006 | 0.055 | 0.078 |
|  | FBG | 0.064 | 0.004 | 0.057 | 0.071 |
|  | HDL_C | 0.060 | 0.004 | 0.053 | 0.069 |
|  | WBC | 0.050 | 0.004 | 0.042 | 0.058 |
|  | SIRI | 0.042 | 0.006 | 0.032 | 0.054 |
| Class 2 | METS_IR | 0.783 | 0.023 | 0.741 | 0.826 |
|  | HSI | 0.731 | 0.020 | 0.693 | 0.773 |
|  | MONO | 0.360 | 0.010 | 0.341 | 0.380 |
|  | ALT | 0.346 | 0.011 | 0.324 | 0.369 |
|  | SUA | 0.201 | 0.008 | 0.186 | 0.217 |
|  | WBC | 0.119 | 0.005 | 0.109 | 0.128 |
|  | TG | 0.115 | 0.004 | 0.106 | 0.124 |
|  | Hypertension | 0.013 | 0.001 | 0.011 | 0.016 |
| Class 3 | HSI | 0.706 | 0.022 | 0.663 | 0.749 |
|  | METS_IR | 0.415 | 0.016 | 0.384 | 0.447 |
|  | TG | 0.296 | 0.013 | 0.271 | 0.322 |
|  | SUA | 0.262 | 0.011 | 0.241 | 0.284 |
|  | TP | 0.211 | 0.012 | 0.187 | 0.233 |
|  | r_GT | 0.081 | 0.004 | 0.073 | 0.090 |
|  | Hypertension | 0.020 | 0.001 | 0.018 | 0.022 |
| Class 4 | METS_IR | 0.497 | 0.020 | 0.456 | 0.536 |
|  | HSI | 0.272 | 0.013 | 0.246 | 0.297 |
|  | TG | 0.188 | 0.007 | 0.176 | 0.201 |
|  | DBIL | 0.171 | 0.006 | 0.159 | 0.182 |
|  | NEUT | 0.140 | 0.005 | 0.130 | 0.150 |
|  | ALB | 0.133 | 0.004 | 0.125 | 0.141 |
|  | HDL_C | 0.115 | 0.005 | 0.105 | 0.125 |
|  | TBIL | 0.104 | 0.004 | 0.096 | 0.113 |
|  | TP | 0.104 | 0.003 | 0.097 | 0.110 |
|  | eGFR | 0.076 | 0.004 | 0.068 | 0.084 |
|  | HGB | 0.071 | 0.002 | 0.066 | 0.075 |
|  | Hypertension | 0.058 | 0.002 | 0.055 | 0.061 |
|  | FBG | 0.056 | 0.002 | 0.051 | 0.060 |

FBG, fasting blood sugar; glycated hemoglobin, HbA1c; HDL-C, high-density lipoprotein-cholesterol; TG, triglyceride; r-GT, r-Glutamyl Transpeptidase; ALB, albumin; TBIL, total bilirubin; WBC, white blood cell; GLB, globulin; DBIL, direct bilirubin; TP, total protein; SUA, serum uric acid; MONO, monocyte; ALT, alanine aminotransferase; HGB, hemoglobin; eGFR, estimated glomerular filtration rate; NEUT, neutrophil; METS-IR, metabolic score for insulin resistance; SIRI, systemic Inflammatory Response Index; HSI, Hepatic Steatosis Index.
